# Azithromycin Clears *Bordetella pertussis* Infection in Mice but Also Modulates Innate and Adaptive Immune Responses and T Cell Memory

**DOI:** 10.3389/fimmu.2018.01764

**Published:** 2018-07-30

**Authors:** Lisa Borkner, Alicja Misiak, Mieszko M. Wilk, Kingston H. G. Mills

**Affiliations:** Immune Regulation Research Group, School of Biochemistry and Immunology, Trinity Biomedical Sciences Institute, Trinity College Dublin, Dublin, Ireland

**Keywords:** *Bordetella pertussis*, macrolide antibiotic, immune modulation, Th1/Th17 cell, innate immunity, memory T cells, pertussis vaccine

## Abstract

Treatment with the macrolide antibiotic azithromycin (AZM) is an important intervention for controlling infection of children with *Bordetella pertussis* and as a prophylaxis for preventing transmission to family members. However, antibiotics are known to have immunomodulatory effects independent of their antimicrobial activity. Here, we used a mouse model to examine the effects of AZM treatment on clearance of *B. pertussis* and induction of innate and adaptive immunity. We found that treatment of mice with AZM either 7 or 14 days post challenge effectively cleared the bacteria from the lungs. The numbers of innate immune cells in the lungs were significantly reduced in antibiotic-treated mice. Furthermore, AZM reduced the activation status of macrophages and dendritic cells, but only in mice treated on day 7. Early treatment with antibiotics also reduced the frequency of tissue-resident T cells and IL-17-producing cells in the lungs. To assess the immunomodulatory effects of AZM independent of its antimicrobial activity, mice were antibiotic treated during immunization with a whole cell pertussis (wP) vaccine. Protection against *B. pertussis* induced by immunization with wP was slightly reduced in AZM-treated mice. Antibiotic-treated wP-immunized mice had reduced numbers of lung-resident memory CD4 T cells and IL-17-production and reduced CD49d expression on splenic CD4 T cells after challenge, suggestive of impaired CD4 T cell memory. Taken together these results suggest that AZM can modulate the induction of memory CD4 T cells during *B. pertussis* infection, but this may in part be due to the clearance of *B. pertussis* and resulting loss of components that stimulate innate and adaptive immune response.

## Introduction

The Gram-negative, aerobic coccobacillus *Bordetella pertussis* is the causative agent of whooping cough (pertussis), a highly contagious infectious disease of the respiratory tract with high mortality in newborns and infants. While pertussis is a vaccine preventable disease, the incidence of pertussis has been increased in many countries during the last decade, despite high vaccine coverage (1, 2). It has been hypothesized that the resurgence of pertussis reflects ineffective or waning immunity induced by current acellular pertussis (aP) vaccines, as well as the emergence of strains of *B. pertussis* with mutations or deletion of antigens in the aP vaccines (2, 3). The development of more effective vaccines is one solution, however, antibiotic treatment of patients as well as post exposure prophylaxis of family members is at present an important medical intervention.

The antibiotics recommended for the treatment of whooping cough belong to the macrolide class. Among those, azithromycin (AZM) can be effective in a shorter course of treatment, has less gastro-intestinal side effects than other agents (4), and is the macrolide of choice for the treatment of infants younger than 1 month (5). It is paramount to start treatment early in infection during the catarrhal stage to control the bacterial load and reduce symptoms. Antibiotic treatment during the paroxysmal stage has no impact on disease symptoms, but is still important to render patients non-infectious and stop the spread of the infection (6).

Following infection with *B. pertussis* the bacteria are contained by the innate immune system, including cells like neutrophils, macrophages, and dendritic cells (DCs) (7). Bacterial clearance and protection against re-infection, however, is dependent on the adaptive immune system, especially *B. pertussis*-specific CD4 Th1 and Th17 cells, but also opsonizing antibodies (7–9). Furthermore, recent studies in a mouse model of *B. pertussis* have demonstrated a role for CD4 lung tissue-resident memory T (T_RM_) cells in protective immunity against re-challenge with *B. pertussis* (10).

Proliferation of CD4 T cells and their entry into the effector phase is dependent on the continuous presence of cognate antigen for their expansion (11, 12). Studies in an influenza virus infection model have shown that entry of CD4 T cells into the memory phase requires antigen presentation at a memory checkpoint during the effector phase (13). This suggests that induction, maintenance, and memory of the CD4 T cell response may be sensitive to the loss of antigen following antibiotic intervention during an infection. Studies with *Listeria, Salmonella*, and *Chlamydia* have revealed that CD4 T cell memory can be impaired following early treatment with antibiotics (14–16). Furthermore, treatment of mice with ampicillin at 24 h of infection with *Listeria* resulted in the downregulation of CD80, CD86, and MHCII expression on DCs, indicating a reduced activation status of the innate immune system after antibiotic-induced bacterial clearance (16).

Independent of their antimicrobial properties, macrolide antibiotics are known to have immunomodulatory properties, and are used in the treatment of several chronic inflammatory diseases, such as asthma (17, 18), chronic obstructive pulmonary disease (COPD) (19), and non-cystic fibrosis bronchiectasis (20). AZM has been shown to shift the phenotype of macrophages from the inflammatory M1 phenotype toward the anti-inflammatory M2 phenotype (21, 22), to reduce the expression of activation markers CD80 and CD86 on monocyte-derived DCs (mo-DCs) (23), and to suppress CD4 T cell activation (24). While these effects can have a positive effect in chronic inflammatory conditions, they might prove detrimental during the induction of protective immunity against infection. Indeed, it has been reported that treatment of mice with AZM during vaccination with a 7-valent, polysaccharide, pneumococcal conjugate vaccine (PCV7) resulted in a reduction in antigen-specific proliferation of spleen cells and antigen-specific antibody titers and suppression of nasal clearance after bacterial challenge (25).

In this study, we examined the effects of AZM treatment early (day 7) and later (day 14) during *B. pertussis* infection on clearance of the bacteria from the lungs and immune response directed against the bacterium. We found that treatment of mice with AZM either at 7 or 14 day post *B. pertussis* challenge effectively cleared the infection from the lungs. However, early treatment also modulated the innate and adaptive immune response in the lungs, especially affecting the frequency of tissue-resident T cells and IL-17-producing T cells. Treatment of mice with AZM during immunization with a whole cell pertussis (wP) vaccine also inhibited the induction of tissue-resident CD4 T cells and IL-17-production in the lungs and CD49d^+^ CD4 T cells in the spleen after *B. pertussis* challenge. Our results suggest that AZM has immunomodulatory as well as antimicrobial activity, and while effective in clearing the bacteria when administered early in infection with *B. pertussis*, the antibiotic treatment may also blunt the induction of immunological memory.

## Materials and Methods

### Animals

C57BL/6 (8 week old) mice were obtained from Harlan Laboratories UK and housed in a specific pathogen-free facility in the Comparative Medicine Unit, Trinity College Dublin. All animal experiments were conducted in accordance with the recommendations and guidelines and under licenses approved by the Health Products Regulatory Authority of Ireland in accordance and with prior ethical approval from Trinity College Dublin Animal Research Ethics Committee.

### *B. pertussis* Respiratory Challenge

*Bordetella pertussis* bacteria were grown from a frozen stock on Bordet Gengou plates containing glycerol and horse blood (Cruinn) at 37°C. After 3 days of culture, the bacteria were collected in supplemented Stainer-Scholte medium and cultured overnight at 37°C in a shaking incubator at 220 rpm. Bacteria were centrifuged and resuspended in 1% casein solution, and the OD was measured at 600 nm. *B. pertussis* infection of C57BL/6 mice was performed by aerosol challenge (BP338 strain; 1 × 10^9^ CFU/ml) administered using a nebulizer (PARI TurboBOY SX) over 10 min (26). The course of infection was followed by performing CFU counts on lung homogenates at intervals post challenge, as described (26).

### AZM Treatment

Mice (mean bodyweight 20 g) were treated with 100 µl of AZM (20 mg/ml; 100 mg/kg) *via* oral gavage, AZM dihydrate (Sigma) stock suspension (200 mg/ml, in DMSO) was diluted 1:10 in autoclaved drinking water to a final concentration of 20 mg/ml.

### Immunization

Mice were immunized with 1:40 human dose of wP vaccine (National Institute of Biological Standards and Control, South Mimms, UK; NIBSC batch 41S) in 200 µl PBS *via* intraperitoneal injection at 6 and 2 weeks before *B. pertussis* challenge. One group of mice was treated with AZM and received one dose of AZM 1 day before 6 days after the first immunization and 1 day before the second immunization.

### Isolation and FACS Analysis of Cells From Lung or Spleen Tissue

Lung tissue was chopped and digested with collagenase-D (1 mg/ml; Roche) and DNase I (10 mg/ml; Sigma-Aldrich) for 1 h at 37°C with agitation. To detect cytokines, brefeldin A (5 µg/ml) was added during the digest. Lungs or spleens were passed through a 70-µm cell strainer to a obtain single-cell suspension, followed by RBC lysis with ACK buffer. The cells were incubated with Fcγblock (anti-CD16/CD32 antibody, BD Biosciences) (1:100) to block IgG Fc receptors. Cells were incubated with LIVE/DEAD Aqua (Invitrogen), followed by surface staining with fluorochrome-conjugated anti-mouse Abs for various markers. The following surface Abs were used: CD69-FITC, CD3-APC-ef780, MHCII-APC, Ly6C-PerCP-Cy5.5, CD86-FITC, CD11b-APC-ef780, CD38-ef450, F4/80-PE-Cy5, CD49d-PerCP-ef710, CD3-AF700, CD8-APC-ef780, CD44-PE-Cy7, CD4-PE-Cy5 (eBiosciences), CD44-BV605, CD4-BV785, CD103-PE, Ly6G-BV605, CD80-PE-Dazzle594 (BioLegend), CD62L-PE-CF594, SiglecF-PE, CD103-BV786 (BD Biosciences). For detection of intracellular cytokines, cells were fixed in 2% PFA and permeabilized with 0.5% saponin (Sigma-Aldrich, Ireland), followed by staining with IL-17A–V450 (BD Biosciences). Fluorescence minus one samples were used as controls. Flow cytometric analysis was performed on an LSR Fortessa, and data were acquired using Diva software (BD Biosciences). The results were analyzed using FlowJo software (TreeStar).

### Antigen-Specific IL-17 and IFN-γ Production by Spleen Cells

Spleens were passed through a 70-µl cell strainer to obtain a single-cell suspension and were cultured with filamentous hemagglutinin (FHA; 2 µg/ml) or sonicated *B. pertussis* (sBp; 5 µg/ml), or medium only. After 3 days, IFN-γ and IL-17A concentrations were quantified in supernatants by ELISA.

### IgG in Serum

Blood was collected from mice and left at 4°C overnight. Then samples were centrifuged and the serum was collected. Serial dilutions of serum were analyzed on FHA-coated plates *via* ELISA and endpoint titers were determined.

### Statistical Analysis

Statistical analysis was performed using GraphPad Prism. Data were analyzed using two-way ANOVA, followed by the multiple comparison tests, or a two-tailed unpaired *t*-test, as appropriate. Data are expressed as mean with SD and were deemed statistically significant when *p* < 0.05.

## Results

### AZM Treatment Clears *B. pertussis* Infection From the Lungs of Mice Within 3 Days

Azithromycin is a well-established treatment option for *B. pertussis* infection in humans (4, 5). To determine whether AZM is effective at clearing the infection, we used a mouse model where 6- to 8-week-old female C57BL/6 mice were aerosol challenged with *B. pertussis* and treated orally with AZM (100 mg/kg), either 7 or 14 days post challenge (dpc). Days 7 and 14 were chosen because CD4 T cell responses against *B. pertussis* develop rather slowly with a peak in lungs at around 14 dpc (7, 10), compared with the CD4 T cell responses to other bacterial infections such as *Listeria monocytogenes* or *Salmonella*, which peak around 7 dpc or earlier (27, 28). In addition, treatment at day 7 post challenge mirrors treatment during the catarrhal stage (1–2 weeks post infection), while treatment at 14 dpc coincides with the beginning of the paroxysmal stage in patients (5).

**Figure 1:**
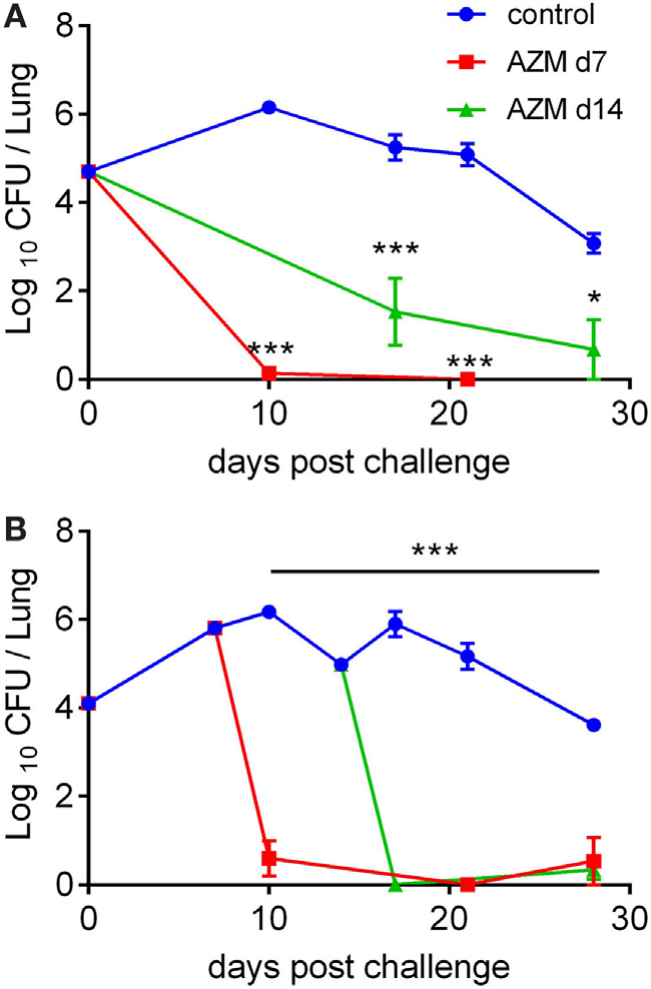
Course of *Bordetella pertussis* infection in azithromycin (AZM)-treated mice. C57BL/6 mice were infected by aerosol challenge with *B. pertussis* and treated with AZM on 7 or 14 days post challenge. The control group of mice received DMSO. The course of infection following a high **(A)** and a low **(B)** initial infection dose. The results are mean (±SEM) CFUs on lung homogenates for five mice per group at each timepoint. Statistical significances were determined *via* two-way ANOVA followed by Sidak’s multiple comparisons test, *p < 0.05, ***p < 0.001.

Analysis of CFUs in the lungs revealed that treatment of mice with AZM at 7 dpc led to complete clearance of bacteria as early as 3 days after treatment, regardless of whether the initial infection dose was high or low (Figures 1A,B). When the antibiotic was administered at 14 dpc, it eradicated the bacteria from the lungs by day 3 post-treatment if the infection dose was low (Figure 1B), but was slightly less effective in clearing a higher infection dose (Figure 1A). These results show that AZM given orally once in a high dose is very efficient at clearing *B. pertussis* from the lungs in the mouse model, especially when administered early.

### Treatment With AZM Reduces the Infiltration of Innate Immune Cells Into the Lungs

**Figure 2:**
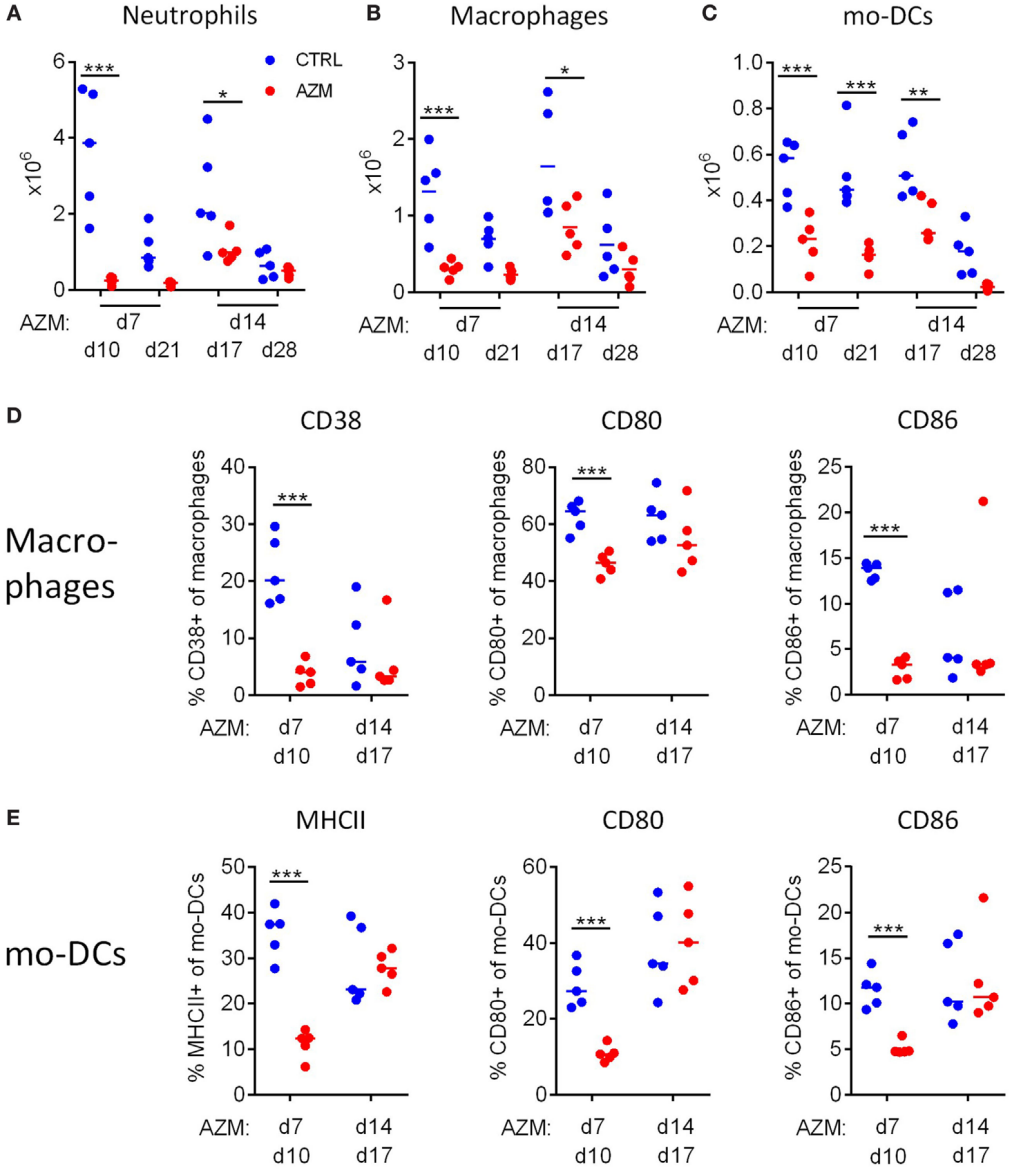
Azithromycin (AZM) treatment early in infection modulates innate immune responses in the lungs. Innate immune cell composition in lung digests were characterized by flow cytometry and absolute cell counts were calculated. Neutrophils were described as SiglecF^−^ B220^−^ Ly6C^+^ Ly6G^+^ **(A)**, macrophages as SiglecF^−^ B220^−^ Ly6C^−^ Ly6G^−^ CD11b^+^ F4/80^+^ **(B)**, and monocyte-derived DCs (mo-DCs) as SiglecF^−^ B220^−^ Ly6C^−^ Ly6G^−^ CD11b^+^ F4/80^−^ **(C)**. Surface expression of activation markers CD38, CD80, and CD86 on macrophages **(D)** and MHCII, CD80, and CD86 on mo-DCs **(E)** were analyzed 3 days after treatment. Results for five mice per group at each timepoint, horizontal lines indicate mean. Significances were determined *via* **(A–C)** two-way ANOVA followed by Sidak’s multiple comparisons test, **(D,E)** *t*-test, *p < 0.05, **p < 0.01, ***p < 0.001.

We assessed the impact of AZM-mediated bacteria clearance on the innate immune response in the lungs. Infected mice were treated with AZM 7 or 14 dpc and were sacrificed 3 or 14 days post-treatment. Lung mononuclear cells were recovered and analyzed by flow cytometry, using the gating strategy shown in Figure S1 in Supplementary Material. We observed a significant reduction of the total numbers of infiltrating neutrophils (SiglecF^−^ B220^−^ Ly6C^+^ Ly6G^+^) (Figure 2A), macrophages (SiglecF^−^ B220^−^ Ly6C^−^ Ly6G^−^ CD11b^+^ F4/80^+^) (Figure 2B), and mo-DCs (SiglecF^−^ B220^−^ Ly6C^−^ Ly6G^−^ CD11b^+^ F4/80^−^) (Figure 2C) in the lungs of AZM-treated mice. This was highly significant for treatment at 7 dpc, and the loss was still substantial following treatment at 14 dpc. However, only early treatment had an impact on the activation status of the innate immune cells. The expression of the M1 macrophage marker CD38 as well as the activation markers CD80 and CD86 were significantly reduced on macrophages 3 days after treatment on 7 dpc (Figure 2D). We could not find any expression of the M2 marker CD206 on macrophages of infected mice, neither in the untreated control nor AZM-treated mice. Similarly, MHCII, CD80, and CD86 expression on mo-DCs was reduced 3 days after early treatment with AZM (Figure 2E). While some activation markers (CD38 and CD86 on macrophages) were already reduced in untreated *B. pertussis* infected mice at 17 dpc (3 days post-treatment at 14 dpc), CD80 on macrophages and mo-DCs and MHCII and CD86 on mo-DCs were still highly expressed. In neither case did AZM treatment lead to a decrease in the expression of activation markers to the levels observed in untreated mice. These findings suggest that while eradication of the bacteria is sufficient to reduce the infiltration of innate immune cells into the lung in general, only early intervention has an impact on the activation status of those cells.

### AZM Treatment at 7 dpc Reduces the Induction of CD4 T Cell Responses

**Figure 3:**
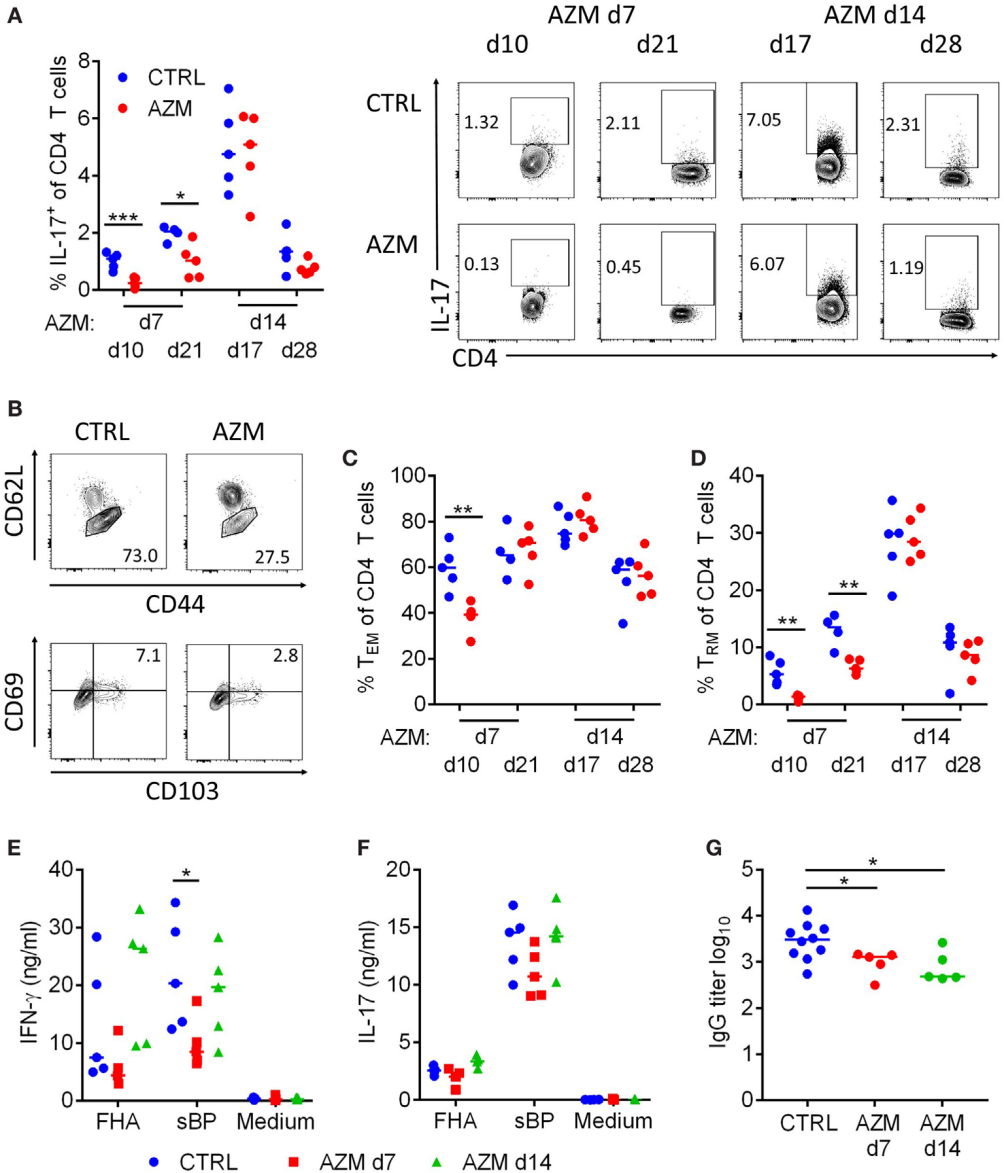
Azithromycin (AZM) treatment at 7 days post *Bordetella pertussis* challenge modulates induction of T cell and antibody responses. CD4 T cells in lung digests were characterized by flow cytometry. The percentage of IL-17-producing cells in the CD4 population was analyzed *via* intracellular cytokine staining 3 and 14 days after treatment. Representative dot plots of the CD4^+^ population are shown **(A)**. T_EM_ (CD62L^low^ CD44^+^) **(C)** and tissue-resident memory T (T_RM_) (CD62L^low^ CD44^+^ CD69^+^ CD103^+^) **(D)** were analyzed 3 and 14 days after treatment. **(B)** Upper panels show representative dot plots of the CD4^+^ population on day 10, lower panels show representative dot plots of the T_EM_ population on day 10. Splenocytes were obtained 28 days post challenge (dpc) and stimulated with filamentous hemagglutinin (FHA) or sBP for 72 h. IFN-γ **(E)** and IL-17 **(F)** production in the supernatants were determined *via* ELISA. Serum was collected from mice on 28 dpc and FHA-specific total IgG serum titers were determined *via* ELISA **(G)**. **(A,C–G)** Horizontal lines indicate mean. Significances were determined *via t*-test, *p < 0.05, **p < 0.01, ***p < 0.001.

Th1 and Th17 CD4 T cells play an important role in the protective immune response and in the establishment of immunological memory against *B. pertussis* (7–10). We therefore investigated whether cellular immune responses to *B. pertussis* were affected by antibiotic treatment. During acute *B. pertussis* infection T cell responses in the lungs peak around 14 dpc. We found a significant reduction in the percentage of IL-17-producing CD4 T cells 3 and 14 days post-treatment on dpc 7 (Figure 3A). The significantly increased production of IL-17 on 17 dpc in the untreated *B. pertussis* infected mice correlates with the peak CD4 T cell response at 14 dpc and is consistent with a previous report (29). Analysis of the percentage of CD62L^low^ CD44^+^ cells (Figure 3B) suggested a reduction in the induction of T_EFF_/T_EM_ cells 3 days but not 14 post-treatment on 7 dpc (Figure 3C). However, the percentage of CD62L^low^CD44^+^CD69^+^CD103^+^ T_RM_ cells (Figure 3B) was significantly reduced on day 10 and 21 in the mice treated 7 dpc (Figure 3D). Administration of AZM on 14 dpc had no effect on the percentage of T_EM_ and T_RM_ within the CD4 T cell compartment. This suggests that by 14 dpc the induction of the CD4 T cell memory is completed and loss of antigen is no longer detrimental.

In parallel with assessment of local immune response in lungs, we examined antigen-specific immune responses to *B. pertussis* in the spleen. Spleen cells from mice 21 dpc that had been treated with AZM on day 7 or 14 were stimulated with either FHA or sBP for 72 h. Analysis of cytokines in the supernatants by ELISA showed a significant reduction of IFN-γ production by spleen cells from mice treated on 7 but not 14 dpc (Figure 3E). IL-17 production was also reduced though not significantly (Figure 3F). These data suggest that eradication of *B. pertussis* and subsequent loss of antigen has a negative effect on the overall CD4 T cell response and the development of CD4 T cell memory, especially when the bacteria are cleared early during the induction of the response. Once the CD4 T cell response is established, persistence of antigen is less important to maintain the T cell response.

### AZM Treatment Impairs Serum Antibody Responses

Antibiotic treatment of bacterial infections has been shown to affect all arms of the adaptive immune response, including the antibody response (16). Therefore, we analyzed FHA-specific serum antibody responses in AZM-treated compared with untreated mice on dpc 28. We found significantly reduced FHA-specific IgG titers in mice that received AZM, regardless of the time point of the intervention (Figure 3G). This indicates that B cell responses are induced by 14 dpc and therefore sensitive to loss of antigens and pathogen-associated molecular patterns (PAMPs) following antibiotic treatment.

### Treatment of Mice With AZM at the Time of Immunization Modulates Protective Immunity Induced With a wP Vaccine

**Figure 4:**
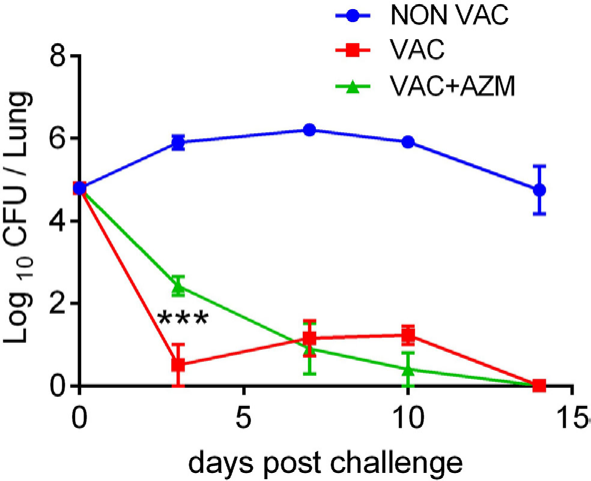
Azithromycin (AZM) treatment during immunization attenuates the protective efficacy of a whole cell pertussis (wP) vaccine. Mice were treated with AZM or vehicle during immunization with wP vaccine 6 and 2 weeks before aerosol challenge with *Bordetella pertussis*. Results are mean ± SEM, CFUs in lung homogenates of four mice per group at each timepoint. Statistical significances were determined *via* two-way ANOVA followed by Tukey’s multiple comparisons test, only VAC compared to VAC + AZM is indicated, significances for comparison between NON VAC and VAC/VAC + AZM was highly significant (***, not indicated), ***p < 0.001.

The results have demonstrated that AZM treatment is effective at clearing the infection, but also suggest that the antibiotic may modulate the generation of innate and adaptive immune responses to the bacteria. However, it is not clear whether this solely reflects loss of bacterial antigens and PAMPs as a result of a lower bacterial load in the lungs or is due to direct modulation of immune cell function by the antibiotic. In order to devolve the antimicrobial from the immunomodulatory effects of AZM, we treated mice with AZM during immunization with a wP vaccine. Mice were treated with AZM (100 mg/kg BW) 1 day prior to and 6 days after i.p. immunization with wP. Mice were given a booster dose of vaccine 4 weeks later and treated with AZM 1 day before immunization. Two weeks after the second immunization, the mice were challenged with *B. pertussis*. Mice immunized with the wP vaccine rapidly cleared the bacteria from their lungs. While AZM-treated wP-immunized mice also cleared the infection, there was significantly higher CFU counts on day 3 when compared with untreated wP-immunized mice (Figure 4). These data suggest that AZM may have a negative impact on the induction of immune responses that mediate immunity induced by wP vaccination.

### AZM Treatment During wP Vaccination Reduces CD4 T_RM_ Cells and IL-17-Producing CD4 T Cells in Lung Post Challenge

**Figure 5:**
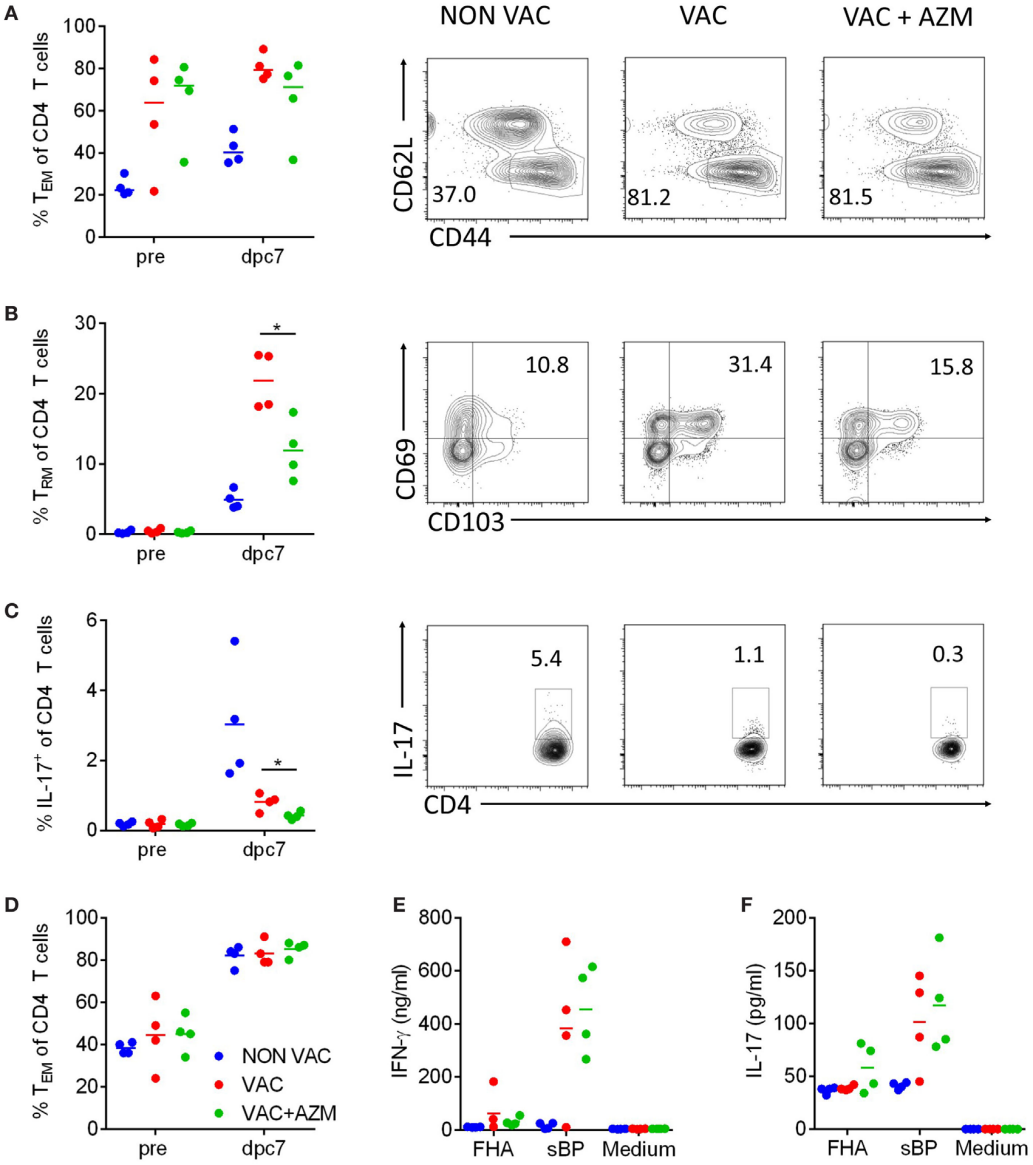
Azithromycin (AZM) treatment during immunization modulates CD4 T cell responses. Mice received AZM or vehicle during immunization with whole cell pertussis vaccine 6 and 2 weeks before aerosol challenge with *Bordetella pertussis*. CD4 T cells were analyzed in lung homogenates 1 day before (pre) and 7 days post challenge (dpc) *via* flow cytometry. The percentage of T_EM_ (CD62L^low^CD44^+^) **(A)**, tissue-resident memory T (T_RM_) (CD62L^low^CD44^+^CD69^+^CD103^+^) **(B)**, and IL-17-producing cells **(C)** in the CD4 population in the lung were determined. Representative dot plots of the CD4^+^ population **(A,C)** and the T_EM_ population **(B)** are shown. Splenocytes were generated 1 day before (pre) and on 7 dpc and analyzed *via* flow cytometry. The percentage of T_EM_ cells **(D)** within the CD4 population was determined. Splenocytes were stimulated with filamentous hemagglutinin (FHA) or sBP for 72 h. IFN-γ **(E)** and IL-17 **(F)** production in the supernatants were determined *via* ELISA. Four mice per group and timepoint, horizontal lines indicate mean. Significances were determined *via t*-test comparing the VAC and VAC + AZM group, *p < 0.05.

Analysis of the CD4 T cell compartment in the lung and spleen pre-challenge and 7 dpc showed higher numbers of CD4 T cells in immunized compared with non-immunized mice, but there was no difference in the absolute number of CD4 T cells induced by the vaccine in AZM-treated compared with untreated mice (data not shown). Furthermore, AZM treatment had no impact on the frequency of CD44^+^CD62L^−^ cells in the lungs (Figure 5A). However, further analysis of memory T cells in the lungs revealed that the expansion of T_RM_ cells after *B. pertussis* challenge was affected by treatment with AZM during immunization with wP. T_RM_ cells were enhanced in the lung 7 dpc in mice immunized with wP vaccine and this increase was significantly reversed in AZM-treated vaccinated mice (Figure 5B). Furthermore, IL-17-production by CD4 T cells was significantly lower in AZM-treated compared with untreated mice (Figure 5C). The lower frequency of IL-17-producing CD4 T cells in immunized compared with non-immunized mice 7 dpc probably reflects the lower bacteria load in these animals at this time point. While the local immune response in the lungs of AZM-treated mice was significantly impaired, we did not find any reduction in T_EM_ cells in the spleen (Figure 5D) or antigen-specific IFN-γ or IL-17 production by spleen cells in response to *in vitro* re-stimulation with sBP or FHA; strong Th1 and Th17 type responses were observed in wP-vaccinated mice with or without AZM treatment (Figures 5E,F). These data demonstrated that AZM can modulate expansion of memory T cells, especially those that secrete IL-17, in the lung post *B. pertussis* challenge in wP-immunized mice but has little effect on T cell responses in the periphery.

### AZM Treatment During Vaccination Leads to Reduced CD49d Expression on CD4 T Cells in Spleen After Challenge

**Figure 6:**
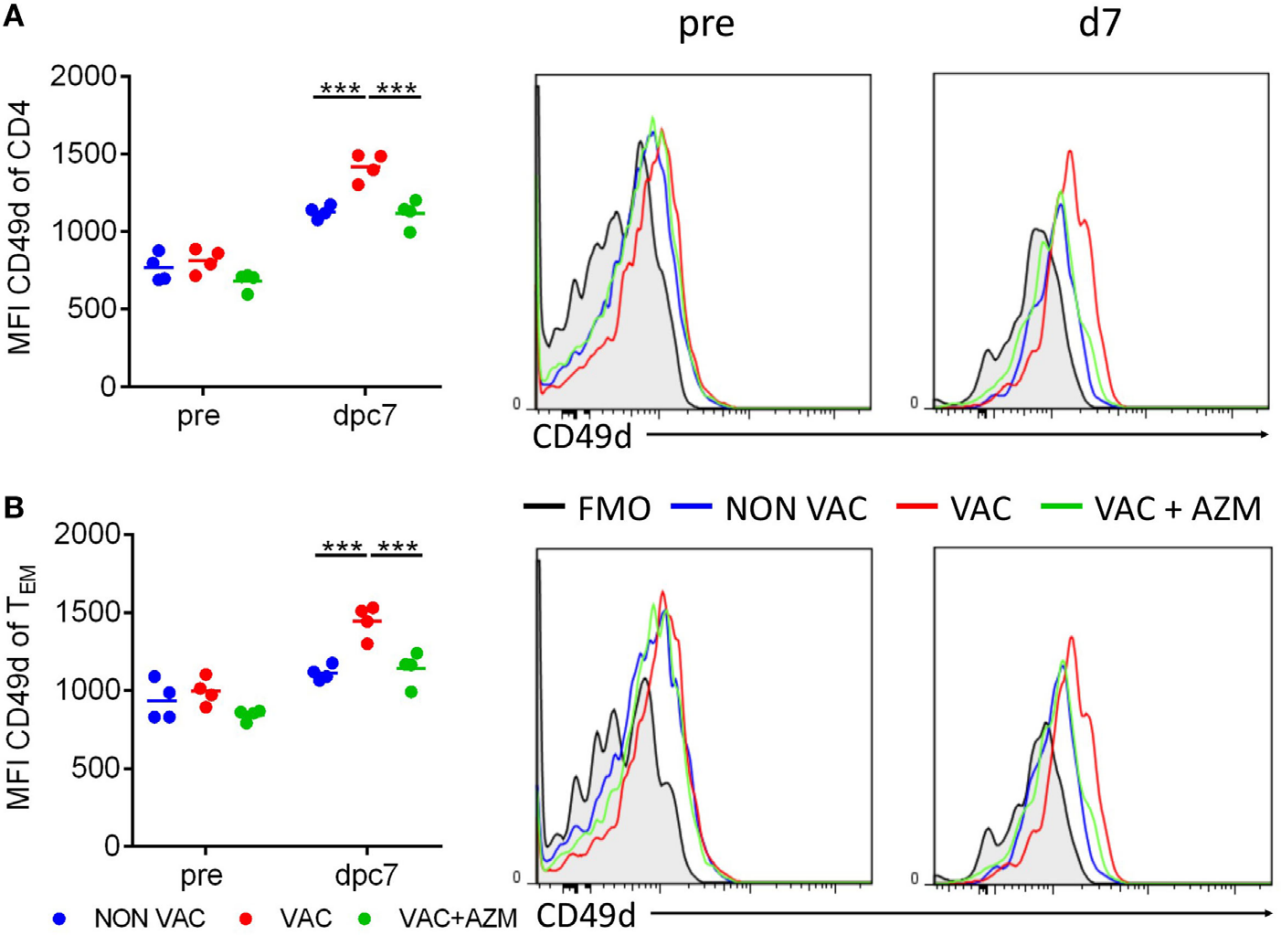
Azithromycin treatment during immunization leads to reduced CD49d expression on splenic CD4 T cells. Mice received azithromycin or vehicle during immunization with whole cell pertussis vaccine 6 and 2 weeks before aerosol challenge with *Bordetella pertussis*. CD49d expression on CD4 T cells **(A)** and CD4 T_EM_ T cells **(B)** in the spleen were analyzed 1 day before (pre) and 7 dpc *via* flow cytometry.

Effective immune responses at mucosal surfaces are dependent on T cell activation, migration, and adhesion at the site of infection. The integrin α4β1 (VLA4) is a mucosal-associated trafficking receptor that facilitates lymphocyte migration to the lungs (29). CD49d, the α4 chain of α4β1, is also involved in T cell co-stimulation. It has been reported that *B. pertussis* can inhibit the expression of α4 integrins on CD4 T cells, which delayed CD4 T cell infiltration into the lungs (30). When we examined CD49d expression on CD4 T cells, we found that expression of this integrin was enhanced on CD4 T cells in the spleen of wP-vaccinated mice post challenge and this was significantly impaired in AZM-treated mice (Figure 6A). We also found that CD49d expression was enhanced on CD4 T_EM_ cell in the spleen of mice immunized with wP and this was reversed in wP-immunized mice treated with AZM (Figure 6B). When taken together with our demonstration of impaired T_RM_ cells in the lungs (Figure 5), these findings suggest that AZM may modulate expansion and migration of antigen-experienced CD4 T cells from the periphery to the lungs by suppressing expression of α4 integrins.

## Discussion

The significant findings of this study are that treatment of mice with the macrolide antibiotic AZM effectively clears an infection of *B. pertussis*, especially when administered early after respiratory challenge (day 7). The antibiotic-mediated clearance of the bacteria was associated with a reduction in local innate and adaptive immune responses in the lungs and this probably reflects the reduced antigen load and reduced PAMPs that stimulate innate immune responses. However, studies in wP-immunized mice revealed that AZM can also modulate immune response to *B. pertussis*, attenuating the induction of IL-17-secreting T cells and the generation of T cell memory.

Pertussis is a severe respiratory disease with high mortality in newborns and infants, and despite good vaccine coverage, the incidence in adults, adolescents, as well as in children has increased during the last decade, requiring treatment with antibiotics to control the disease and limit the spread of the infection (1, 2). AZM, an antibiotic of the macrolide class, is effective in the treatment of pertussis, but only when given in the catarrhal but not in the paroxysmal stage (4–6). Our data show that AZM is highly effective in clearing the infection when given at 7 dpc, reducing the bacterial load in the lungs to undetectable levels within 3 days, but is less effective when given at 14 dpc. This may reflect the increased number of bacteria occupying intracellular niches, such as macrophages (31) by 14 dpc, allowing them to survive longer in the presence of AZM.

Azithromycin has immunomodulatory properties independent of its antimicrobial function that are exploited in the treatment of chronic inflammatory diseases, such as asthma (17, 18), COPD (19), and non-cystic fibrosis bronchiectasis (20). In general, AZM promotes an anti-inflammatory environment by shifting macrophages from the inflammatory M1 toward the anti-inflammatory M2 phenotype (21, 22), by reducing the activation of mo-DCs as well as their stimulatory effect on CD4 T cells *in vitro* (23), and by directly suppressing CD4 T cell activation through inhibition of mTOR activity (24). We found that treatment of mice with AZM 7 or 14 dpc with *B. pertussis* significantly reduced the number of neutrophils, macrophages, and mo-DCs in the lungs. Furthermore, expression of activation markers CD38, CD80/86, and MHC class II was reduced on macrophages and mo-DCs after treatment on 7 dpc. However, this was not observed at 14 dpc, suggesting that the loss of innate activation in mice treated with AZM at 7 dpc may reflect a reduction in bacterial PAMPs through clearance of *B. pertussis* form the lungs.

Antibiotic treatment of bacterial infection has also been shown to impair induction of T cell memory, especially when treatment is initiated early in infection (14–16). In the *B. pertussis* mouse model, we found impaired CD4 T cell function and memory in mice treated with AZM. Mice treated with AZM at 7 dpc had reduced numbers of IL-17-producing CD4 T cells and CD4 T_RM_ cells in the lungs. Furthermore, antigen stimulated spleen cells from mice treated 7 dpc produced less IFN-γ and IL-17, whereas mice treated 14 dpc had similar antigen-specific responses to those seen in untreated infected mice. These findings are consistent with reports that CD4 T cells require a prolonged period of antigen presentation for activation (11, 12). They also provide further evidence that AZM-mediated attenuation of the immune responses to *B. pertussis* may at least in part reflect the reduced load of bacterial antigens in the lungs of antibiotic-treated mice. The reduced activation status of antigen-presenting cells after AZM treatment at 7 dpc might also impair T cell activation. It has been reported that human mo-DCs induced *in vitro* in the presence of AZM have reduced activation and capacity to induce T cell proliferation (23). However, a loss of PAMPs due to antimicrobial activity of AZM is a more likely explanation for the reduced T cells activation that we observed in our *in vivo* experiments with *B. pertussis* infection. Nevertheless, reduced T cell activation may reflect direct immunomodulation by AZM as well as reduced activation of antigen-presenting cells through a reduction in PAMPs.

Although our data suggest that the immunomodulatory effects of AZM may be secondary to its antimicrobial activity and clearance of the infection, we also demonstrate that the antibiotic can more directly exert immunomodulatory effects *in vivo*. Evidence for this was provided by experiments where mice were immunized with wP with or without treatment with AZM at the time of immunization. We found a significant reduction in CD4 T cells responses and clearance of the bacteria from the lungs of AZM-treated compared with non-treated mice immunized with the wP vaccine. This is consistent with a study that reported that AZM treatment impaired immune protection in a mouse model for lung infection with *Streptococcus pneumoniae* after immunization with a 7-valent polysaccharide pneumococcal conjugate vaccine (PCV7) (25).

We found that treatment of mice with AZM during immunization with a wP vaccine had a negative effect on the expansion of splenic antigen-specific CD4 T cells after challenge. The enhanced expression of CD49d, the α4 chain of VLA4, on CD4 T cells post *B. pertussis* challenge of wP-immunized mice was reversed by treatment with AZM. The integrin VLA4 has been shown to be important for T cell migration into lungs during *B. pertussis* infection (30). CD49d has also been identified as a marker for antigen-specific CD4 T cells and it has been shown that only CD49d^+^ CD4 T cells undergo rapid proliferation post re-challenge in a LCMV model (32). In addition, it has been shown that AZM treatment during PVC7 vaccination led to reduced antigen-specific proliferation of spleen cells (25).

Our data show that early treatment of *B. pertussis* infected mice with AZM effectively clears the infection, but this can impair the activation of innate and adaptive immune response to *B. pertussis* and the induction of CD4 T cell memory. Delaying the AZM treatment to day 14 of infection was also effective at clearing the infection, though not as efficiently with a high challenge dose, this did result in less marked modulation of the immune response to *B. pertussis*. However, delaying antibiotic treatment, as it has been suggested for other bacterial infections (16), may not be an option in vulnerable infants infected with *B. pertussis*. Furthermore, our study suggests that AZM treatment may blunt the induction of long-term protective immunity induced by natural infection with *B. pertussis*. The findings also suggested that sustained activation of innate and adaptive immune responses by PAMPs and antigen, respectively, for up to 14 days may be required for optimal induction of immunological memory. Finally, our findings have implications for antibiotic treatment of human volunteers exposed to *B. pertussis* in a human challenge model under development (33) and provide comfort that treatment with a single oral dose of macrolide antibiotics is an effective means of terminating the infection provided they are administered 1–2 weeks after the intranasal challenge with live *B. pertussis*.

## Ethics Statement

All animal experiments were conducted in accordance with the recommendations and guidelines and under licenses approved by the Health Products Regulatory Authority of Ireland in accordance and with prior ethical approval from Trinity College Dublin Animal Research Ethics Committee.

## Author Contributions

LB designed and performed experiments, analyzed data, and co-wrote the manuscript. AM performed experiments. MW contributed ideas and assisted in the development of methodologies. KM conceived ideas, oversaw the project, and co-wrote the manuscript.

## Conflict of Interest Statement

The authors declare that the research was conducted in the absence of any commercial or financial relationships that could be construed as a potential conflict of interest.

## Supplementary Material

Figure S1 Gating strategy for innate immune cell populations. Cells generated by lung digest were antibody stained and analyzed *via* flow cytometry. After excluding air bubbles at the end of the sample read with a time gate **(A)**, followed by exclusion of doublets and conglomerates **(B)**, and cell debris **(C)**, live cells were determined using a live/dead stain **(D)**. In the live population, cells were first gated on alveolar macrophages (Siglec F^+^ CD11c^+^) and eosinophils (Siglec F^+^ CD11c^low^) and the remaining Siglec F^−^ cells **(E)**. In the Siglec F^−^ population, B cells were excluded as B220^+^ MHCII^+^ **(F)** and from the non-B cell population neutrophils were determined as Ly6G^+^ Ly6C^+^ **(G)**. The non-neutrophil population was split into CD11b^+^ and CD11b^−^ cells **(H)**. The CD11b^+^ population was split into macrophages (CD11b^+^ F4/80^+^) and monocyte-derived DCs (CD11b^+^ F4/80^−^) **(I)**.
