# Supplementary figures and images for: Azithromycin Clears Bordetella pertussis Infection in Mice but Also Modulates Innate and Adaptive Immune Responses and T Cell Memory
Source: Front Immunol. 2018 Jul 30;9:1764. doi: 10.3389/fimmu.2018.01764 (PMC6077268; doi:10.3389/fimmu.2018.01764)

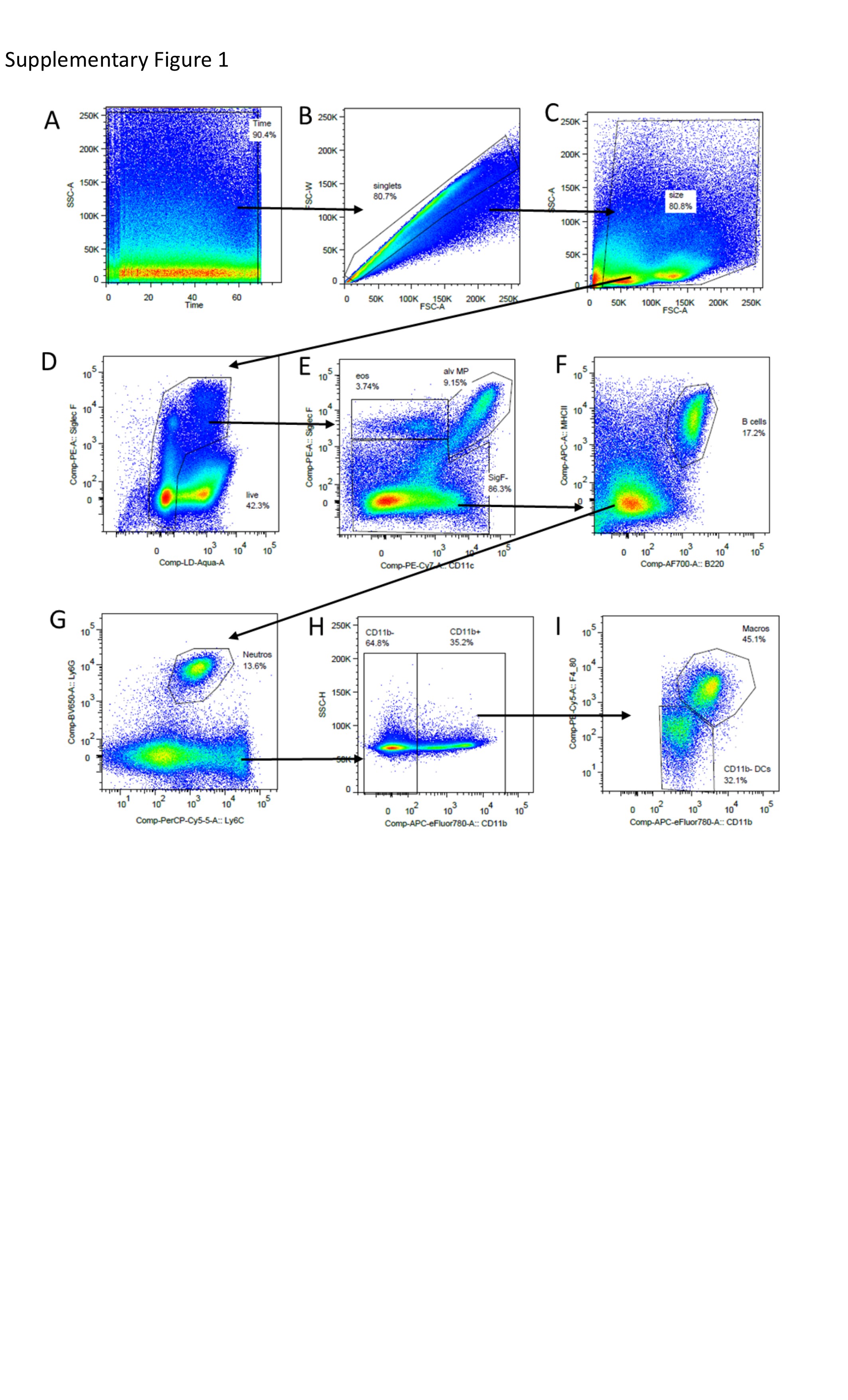

Supplement: Figure S1 — Gating strategy for innate immune cell populations. Cells generated by lung digest were antibody stained and analyzed via flow cytometry. After excluding air bubbles at the end of the sample read with a time gate (A), followed by exclusion of doublets and conglomerates (B), and cell debris (C), live cells were determined using a live/dead stain (D). In the live population, cells were first gated on alveolar macrophages (Siglec F+ CD11c+) and eosinophils (Siglec F+ CD11clow) and the remaining Siglec F− cells (E). In the Siglec F− population, B cells were excluded as B220+ MHCII+ (F) and from the non-B cell population neutrophils were determined as Ly6G+ Ly6C+ (G). The non-neutrophil population was split into CD11b+ and CD11b− cells (H). The CD11b+ population was split into macrophages (CD11b+ F4/80+) and monocyte-derived DCs (CD11b+ F4/80−) (I). [file image_1.jpeg]
